# Identification and Expression Analysis of an Atypical Alkaline Phosphatase in *Emiliania huxleyi*

**DOI:** 10.3389/fmicb.2018.02156

**Published:** 2018-09-19

**Authors:** Tangcheng Li, Chentao Guo, Yaqun Zhang, Cong Wang, Xin Lin, Senjie Lin

**Affiliations:** ^1^State Key Laboratory of Marine Environmental Science and Xiamen City Key Laboratory of Urban Sea Ecological Conservation and Restoration, Xiamen University, Xiamen, China; ^2^Department of Marine Sciences, University of Connecticut, Groton, CT, United States

**Keywords:** *Emiliania huxleyi*, alkaline phosphatase, gene expression, phosphorus limitation, cofactor

## Abstract

*Emiliania huxleyi*, a cosmopolitan coccolithophore in the modern ocean, plays an important role in the carbon cycle and local climate feedback as it can form extensive blooms, calcify, and produce dimethylsulfoniopropionate (DMSP) leading to the generation of dimethyl sulfide (DMS) which affects climate when oxidized in the atmosphere. It is known to be able to utilize dissolved organic phosphorus (DOP) by expressing a specific type of alkaline phosphatase (EHAP1) under phosphorus-limited conditions. In this study, we identified a new alkaline phosphatase (EH-PhoA^aty^) in this species, which we found belongs to the newly classified PhoA^aty^ family. The expression of this atypical phosphatase was up-regulated under P-depleted conditions at both the transcriptional and translational levels, suggesting that *E. huxleyi* is able to express this AP to cope with phosphorus limitation. Comparative analysis revealed different transcriptional expression dynamics between *eh-PhoA*^aty^ and *ehap1*, although both genes exhibited inducible expression under phosphate deficiency. In addition, after AP activity was eliminated by using EDTA to chelate metal ions, we found that AP activity was recovered with the supplement of Ca^2+^ and Zn^2+^, indicative of the adoption of Ca^2+^ as the cofactor under Zn-P co-limited conditions, likely a result of adaptation to oceanic environments where Zn^2+^ is often limiting.

## Introduction

Phosphorus (P) is an essential nutrient required by living cells to synthesize vital biomolecules, such as lipids, nucleic acids, ATP, and signaling molecules (Dyhrman et al., 2012; Karl, 2014; Lin et al., 2016; Luo et al., 2017). In the ocean, phosphorus is one of the major nutrients required for primary production existing in both inorganic and organic forms (Karl, 2000). The preferred form of dissolved inorganic phosphorus (DIP), which can be utilized directly by phytoplankton, is chronically low in many parts of the ocean and seasonally limited in coastal waters (Lomas et al., 2004; Thingstad et al., 2005; Moutin et al., 2007; Van Mooy et al., 2009). In contrast, dissolved organic phosphorus (DOP) is usually more abundant than DIP in the euphotic zone, and mainly comprising phosphoesters (>75%) and phosphonates (nearly 25%) (Clark et al., 1998; Kolowith et al., 2001; Karl and Björkman, 2002). Studies so far have consistently indicated that DOP can be utilized by marine phytoplankton to support primary production. Marine microorganisms have developed various mechanisms to hydrolyze DOP and release inorganic phosphate (P*i*) to meet their P requirements for growth (Dyhrman et al., 2007). Alkaline phosphatase (AP) is the most common DOP hydrolase expressed by marine unicellular microorganisms (Labry et al., 2005; Nicholson et al., 2006; Huang et al., 2007; Duhamel et al., 2010).

AP (EC 3. 1. 3. 1) is a phosphoester hydrolase with two properties: low substrate specificity and an alkaline pH optimum (Masahiro and Kimitoshi, 1998; Lee et al., 2015). In seawater, AP can enable bacteria and phytoplankton to scavenge phosphorus from various chemical forms of DOP when DIP is depleted, although recent studies showed utilization of glucose-6-phosphate and ATP in the dinoflagellate *Karenia mikimotoi* was AP-independent (Luo et al., 2017; Zhang et al., 2017). Because it is inducible by P deficiency, AP activity has been widely accepted as a biomarker of P-stress in phytoplankton (Cembella et al., 1984; Mahaffey et al., 2014). Many studies have been conducted to identify and characterize AP genes in marine bacteria, resulting in the recognition of three homologs, PhoA, PhoX, and PhoD (Gomez and Ingram, 1995; Kriakov et al., 2003; Majumdar et al., 2005). They were found to show different substrate preferences, different ionic activators (Zn^2+^, Mg^2+^, Ca^2+^, Fe^3+^), and different subcellular localizations (Luo et al., 2009; White, 2009). Sharing little sequence similarity with the classified APs (PhoA, PhoD, and PhoX), several distinct types of APs have been identified in the haptophyte *Emiliania huxleyi* (Xu et al., 2006), the pelagophyte *Aureoumbra lagunensis* (Sun et al., 2012), some dinoflagellates (Lin et al., 2011, 2012a,b), and the diatom *Phaeodactylum tricornutum* (Bowler et al., 2008). Among these, AP identified in dinoflagellates has recently been classified as an atypical group of APs (PhoA^aty^), which shares conserved motifs with various putative *PhoA*^aty^ genes in other phytoplankton genomes, including one from *E. huxleyi* (Lin X. et al., 2015).

*Emiliania huxleyi* (Lohman), a cosmopolitan coccolithophore in the modern ocean, forms extensive blooms in both coastal and open oceanic waters (Brown and Yoder, 1994; Paasche, 2002). The blooms of *E. huxleyi* have significant biogeochemical implications, particularly in the global carbon and sulfur cycles through their production of calcite coccoliths and dimethy1sulfoniopropionate (DMSP), the precursor of the climate-relevant gas dimethyl sulfide (DMS) (Paasche, 2002; Marsh, 2003; Rost and Riebesell, 2004). Previous studies revealed that there is a large internal P*i* pool and an inducible AP in several strains making *E. huxleyi* particularly well adapted to low phosphate conditions (Riegman et al., 1992; Dyhrman and Palenik, 2003). Furthermore, kinetic analyses have suggested that *E. huxleyi* possesses more than one type of AP (Dyhrman and Palenik, 2003; Shaked et al., 2006). However, to the best of our knowledge, only one kind of AP gene (*ehap1*) has been documented in this species.

In this study, we attempted to obtain molecular evidence that *E. huxleyi* possesses more APs than just *ehap1* and characterized their differential expression patterns. We identified an atypical AP gene (*eh-PhoA*^aty^) in *E. huxleyi* and found that its expression was inducible under P deficiency at both the transcriptional and translational levels. We compared *eh-PhoA*^aty^ and *ehap1* expression patterns following growth in P-depleted and P-replete conditions. To further characterize APs in *E. huxleyi*, we also examined the subcellular localization and affinity for metal ions as cofactors of APs in cells.

## Materials and Methods

### Algal Cultures and P Treatments

*Emiliania huxleyi* (strain PML B92/11, non-axenic strain) was provided by the Collection Center of Marine Algae, Xiamen University China, and was cultured at 20 ± 1°C under a 14 h: 10 h light/dark cycle with a photon flux of 100 μE m^-2^ s^-1^. Cultures were prepared with 0.22_μm pore-size filtered and autoclaved seawater, and an antibiotic cocktail comprising 100 mg/L streptomycin, 100 mg/L kanamycin and 200 mg/L ampicillin (final concentration) to inhibit the growth of bacteria in the culture (Lin S. et al., 2015, Wang et al., 2016). Experimental cultures were set up in 2 L culture flasks for both P-depleted and P-replete conditions, each in triplicate. Algae were grown in f/2 medium (Guillard and Ryther, 1962) modified with vitamins and trace metals supplied in half, and N:P ratio was 150:1 (P-deplete) and 16:1 (P-replete) respectively (Mckew et al., 2015; Ameijeiras et al., 2016). Cell concentrations were monitored daily using a Sedgwick-Rafter counting chamber (Phycotech, St. Joseph, MI, United States). DIP concentration in each culture was also determined daily by filtering 25 mL culture through a 0.22 μm pore-size mixed-cellulose-ester membrane and filtrate to the molybdenum blue inorganic phosphate assay (Timothy et al., 1984).

### AP Activity Quantification and Subcellular Localization

Bulk AP activity was measured by adding 50 μL of 20 mM *p*-nitro-phenylphosphate (*p*-NPP; prepared in 1 M Tris buffer at pH 9.0) into 1 mL culture sample, followed by 2 h-incubation at 20°C in the dark (Xu et al., 2006; Lin et al., 2011). Samples were then placed on ice to stop further enzymatic activity and centrifuged at 10,000 × *g* for 2 min. The supernatant was transferred into a 96 well plate and the absorbance was measured on a SpectraMax^®^ Paradigm^®^ microplate reader (Molecular Devices, United States) at a wavelength of 405 nm. The absorbance of a dilution series of *p*-NP (the AP-hydrolysis product of *p*-NPP) was used to create a standard curve. AP activity was computed as the amount of *p*-NP produced during the incubation time, based on the absorbance of the test sample and the absorbance-concentration linear regression (standard curve), normalized to per cell and unit time and averaged across triplicate samples.

With this approach, analyses were conducted to partition the AP activity into different compartments of the culture. Besides above mentioned bulk AP activity (W), P-depleted cultures were centrifuged at 4,500 × *g* for 10 min at 20°C and the resulting supernatants were used to determine the activity of secretory AP (S). In parallel, cell pellets were resuspended in autoclaved filtered seawater to determine cell surface AP activity (C), while other replicated cell pellets were homogenized to measure AP activity of cell lysates (CL). To further microscopically examine the subcellular localization of AP in *E. huxleyi*, ELF^®^-97 Phosphatase Substrate (Invitrogen, Carlsbad, CA, United States) was used to label AP in intact cells. Cells were centrifuged at 4,500 × *g* for 10 min. The cell pellets were first incubated in 200 μL 75% (v/v) ethanol for 30 min to remove chlorophyll, and then mixed with ELF^®^-97 phosphatase substrate at a finial concentration of 0.25 mM and incubated for 30 min in the dark (Lin et al., 2012a). Cells were washed twice using sterile seawater and resuspended in 100 μL sterile seawater before microscopic observation. Green fluorescent cell images were taken at different scanning depths using a Laser Scanning Confocal Microscope (LSM780 NLO, excitation: 350–420 nm, ZEISS, Germany), and whole cell images were captured using an epifluorescence microscope (excitation: 300–400 nm, Axio Imager A2, ZEISS corporation, Germany).

### RNA Isolation and cDNA Synthesis

Cells were collected and total RNA was isolated as previously reported (Zhang et al., 2007). Briefly, after cells were homogenized using the Fastprep^®^-24 Sample Preparation System (MP Biomedicals, United States) with bead-beating (0.5 mm mixed 0.1 mm diameter ceramic beads at 5:1), total RNA was extracted using Trizol reagent (Molecular Research Center, Inc., United States) coupled with further purification using Direct-Zol RNA Miniprep (Zymo Research, Orange, CA, United States). The concentration and quality of extracted RNA were determined on a NanoDrop (ND-2000 spectrophotometer; Thermo Scientific, Wilmington, DE, United States). For each sample, 300 ng total RNA was used in cDNA synthesis using the PrimeScriptTM RT reagent Kit (Takara, Clontech, Japan).

### Identification of *eh-PhoA*^aty^ and Computational Prediction of Subcellular Localization

We used the sequence an atypical AP identified in *Amphidinium carterae* as a query to blastx against the genome of *E. huxleyi* strain CCMP1516 (GenBank accession No. GCA_000372725.1). Four hits (*E*-value < 3e-66) from the genome assembly were retrieved and aligned. Conserved regions were identified and used to design degenerate primers (**Table 1**) to obtain homologs in *E. huxleyi* strain PML B92/11. Primers EhuxAP-F4 and EhuxAP-R1 (**Table 1**) were used to amplify the gene fragment of *eh-PhoA*^aty^ from cDNA template of *E. huxleyi* strain PML B92/11. PCR conditions were: 95°C for 3 min followed by 10 cycles of 95°C for 15 s, 52°C for 30 s, 72°C for 1 min, and 20 cycles 95°C for 15 s, 56°C for 30 s, 72°C for 1 min and a final step of extension at 72°C for 5 min. PCR reactions were performed in a total volume of 25 μL, which contained 0.005 U ExTaq HS, 2.5 μL 10 × ExTaq buffer, 0.2 μM of each dNTP, 0.2 μM of each primer. The PCR product with the expected size was purified using the Universal DNA Purification Kit (TransGen, Biotech, Beijing, China) and directly sequenced (BGI, Shanghai, China). Based on the gene fragment obtained, specific primers (**Table 1**) were designed and used to acquire both 5′ and 3′ cDNA ends of the full length ORF region using the SMARTer^®^ RACE 5′/3′ kit (Clontech, Japan). The deduced amino acid sequence of the full length *eh-PhoA*^aty^ was used to conduct a pairwise sequence comparison with two hits acquired from the genome of *E. huxleyi* (CCMP1516) (Accession No: XP 005774892.1 and XP 005761790.1), PhoA^aty^ of dinoflagellate (*Amphidinium carterae*, Accession No: ADT91623.2; *Karenia brevis*, Accession No: AFO84050.1; *Alexandrium tamarense*, Accession No: ALG03341.1; *K. mikimotoi*, Accession No: ALG03306.1), and reported EHAP1 of *E. huxleyi* (CCMP1516) (Accession No: XP 005759684.1, ABI51308.1, XP 005788892.1). Phylogenetic analyses were performed on MEGA v5.5 platform (Tamura et al., 2011), with alignment further visualized using BoxShade^1^ and phylogenetic tree reconstructed by using Neighbor Joining (Saitou and Nei, 1987) and Maximum-Likelihood (Guindon et al., 2010).

**Table 1:** Primers and thermal cycling conditions used in PCRs.

Primer name^∗^	Primer sequence(5′-3′)	Application	Annealing temperature
EhuxAP-F4	TCGAGCCvGAGkmCCTsrCCTGG	AP gene cloning	52 and 56°C
EhuxAP-R1	TGCwCGCyGTTGTGCGmCCACGG	AP gene cloning	52 and 56°C
Ehux3′Race	GATTACGCCAAGCTTGATGAGCATCACCATCGGGTCGAGCGGCG	3′ Race	68° C
Ehux5′Race1	CGCCTCCACGAAG	5′Race	55°C
Ehux5′Race2	ACAGCACACACTATCGATGAGCG	5′ Race	60°C
EhuxRTAP-F2	CGTCATCGACACGAACGAGAC	AP RT-qPCR	55°C
EhuxRTAP-R2	CTCGACCCGATGGTGATGCTC	AP RT-qPCR	55°C
EhuxAPRT-1F	AGCACATGTCGAACCCAA	AP RT-qPCR	55°C
EhuxAPRT-1R	CGCCTCCACGAAGCAG	AP RT-qPCR	55°C
EhuxAPXY-F1	ATGTCGAACCCAAGCGCATACG	AP RT-qPCR	55°C
EhuxAPXY-R1	GTGAGGAGCGAGTCGATCTTGGC	AP RT-qPCR	55°C
Actin-F	TGGATGGTCAAGCTGCTG	AP RT-qPCR	55°C
Actin-R	CATCAAGGAGAAGCTGGC	AP RT-qPCR	55°C


*^∗^F, forward primer; R, reverse primer; v, A, C and G; k, T and G; m, A and C; s, C and G; r, A and G; w, A and T; y, C and T.*

The computational program-CELLO (Yu et al., 2006) which has been used to make protein localization predictions in unicellular organisms (Luo et al., 2009) was used to predict subcellular localizations of APs in *E. huxleyi*. Because no algal model has been built into the program, we applied the plant model in our analysis. Furthermore, signal peptide of APs was determined using SignalP V4.1 (Petersen et al., 2011).

### Real Time Quantitative PCR Analysis of AP Gene Expression

Specific primers (**Table 1**) targeting both *eh-PhoA*^aty^ and *ehap1* (Accession No: XM_005759627.1) were designed respectively for real time quantitative PCR (RT-qPCR) analysis to compare the genes expression in different cultures. RT-qPCR was performed using iQTM SYBR^®^ Green Supermix on a CFX96 Real-time PCR System (Bio-Rad Laboratories, Hercules, CA, United States) essentially following a previously reported protocol (Zhang and Lin, 2003). We used actin as the reference gene because it has been reported to show a relatively stable level of expression (Bach et al., 2013). Purified amplicons for each gene (from a plasmid clone) were diluted to 10^5^-10^10^ copies per reaction to generate standard curves for both the target and the reference genes (Hou et al., 2010). RT-qPCR reactions were carried out in a total volume of 12 μL containing 2.5 μM of each primer, cDNA equivalent to 5 ng of total RNA and 6 μL Supermix. Transcript levels of both test genes were normalized in two ways, to the transcript abundances of the actin gene and to the amount of total RNA used to generate the cDNA template used in the qPCR assay (Cui et al., 2016).

### Western Blot Analysis of EH-PhoA^aty^ Protein Accumulation

A peptide (*p*ACAAP) comprising 180 amino acid residues of ACAAP (amino acid site 220-400), encoded by the gene *acaap* identified in dinoflagellate *A*. *carterae* (Lin et al., 2011) was overexpressed in *E. coli*. Purified *p*ACAAP was used to immunize a rabbit and generate the polyclonal antiserum (Proteintech Group Inc., Wuhan, China). Pairwise comparison showed *p*ACAAP shared sequence similarity of 49% (*E* value = 6e-37) with the counterpart fragments of EH-PhoA^aty^ (amino acid site 196-375). The applicability of this antiserum to determine the EH-PhoA^aty^ expression was verified firstly by the detection of a clear band (∼110 kD) in western blot analysis, which was close to the predicted MW of EH-PhoA^aty^ (**Supplementary Figure 1**). Meanwhile, the two counterpart gel fragments with the range of 100–120 kD and 40–60 kD were cut out from a parallel SDS-PAGE gel, and subjected to mass spectroscopic analysis using a TripleTOF^®^ 5600+ (AB Sciex, United States). We also conducted a competitive immunoreaction with cell free protein of *E. huxelyi* as follows: 5 μL antiserum was pre-incubated overnight with 95 μL antigen (*p*ACAAP) before undertaking western blot analysis; meanwhile, a duplicate blot was immunoreacted with antiserum. This type of competition for the epitope has previously been employed to verify the specificity of antibodies used to detect algal proteins (Lin et al., 1994).

Total proteins were extracted from the P-replete and P-depleted cultures after homogenizing the cells using the Fastprep^®^-24 Sample Preparation System with bead-beating. After centrifugation at 10,000 × *g* for 2 min, the supernatant was transferred into a fresh 1.5 mL tube. Protein concentration was determined using the BCA Protein Assay Kit (TransGen Biotech, Beijing, China) according to the manufacturer’s instructions and absorbance was measured on a SpectraMax^®^ Paradigm^®^ microplate reader (Molecular Devices, United States) at a wavelength of 562-nm (Li et al., 2016). After the protein was denatured at 95°C for 5 min by mixing with β-mercaptoethanol-SDS protein loading buffer (4 folds volume; Solarbio, Beijing, China. Cat. No. P1016), and 15 μg was loaded into each well of a 10% (w/v) SDS-PAGE gel (Bio-Rad, United States). Samples were loaded onto duplicated gels and electrophoresed at 80 V for 30 min then at 120 V for 1 h. The resolved proteins were then transferred to polyvinylidene difluoride (PVDF) membranes (Bio-Rad, Hercules, CA, United States) at 25 V for 30 min using a Trans-Blot SD Semi-Dry Transfer Cell (Bio-Rad, United States). Membranes were subsequently blocked in 5% (w/v) defatted dry milk prepared in Tris buffered saline (TBS) with 0.1 % (v/v) Tween-20 (TBST) over 1 h at room temperature, and incubated with the polyantiserum (diluted 1: 4000 in TBST) and GAPDH (provided by BBI Life Science, Sangon Biotech, Shanghai, China; diluted 1: 1000 in TBST), respectively. The abundance of GAPDH was used as a reference because of the reported relatively stable abundance of this protein in a dinoflagellate (Shi et al., 2015), and the lack of an established reference protein in *E. huxleyi*. After three washes in TBST each for 10 min, the membranes were incubated with a secondary antibody (goat anti-rabbit IgG antibody, TransGen Biotech, Beijing, China; diluted 1: 4000 in TBST) for 1 h. After three washes the membranes were treated with the enhanced chemiluminescent (ECL) substrate (Bio-Rad, Hercules, CA, United States) to detect the immunoreactive bands visualized on the Molecular Imager^®^ Chemi Doc XR system (Bio-Rad, Hercules, CA, United States) and quantified using Image Lab^TM^ software (Li et al., 2016).

### Metal Dependency Analysis of AP Activity in *E*. *huxelyi*

Total proteins were extracted as described above and were subjected to examine the metal dependency of AP in *E*. *huxelyi*. First, a metal chelating reaction was set up in a 96-well plate by mixing 80 μL AP buffer (0.02M Tris-Cl, 0.1M NaCl, pH = 8.0), 5 μL protein, 5 μL EDTA (100 mM) and 5 μL *p*-NPP, and the mix was incubated for 30-min at 20°C in the dark. Then, 10 μL of different metal ions (EDTA, Ca^2+^, Mg^2+^, Zn^2+^, and Co^2+^) were supplied separately into the reaction mix at a final concentration of 10, 10, 10, 8, and 5 mM respectively and incubated at 20°C for another 2 h, each group in triplicate. Meanwhile, the control group was set up with no addition of metal ions. AP activities were measured as described above. Fold change of AP activities of each group was computed as dividing by that of the control group.

### Statistical Analysis

In order to evaluate the statistical significance of the differences observed between the two phosphorus treatments (P-depleted and P-replete groups), a Generalized Linear Model Repeated Measure procedure was applied using SPSS statistic software package, which test the effect of both the treatment factor and treatment-time factors. For comparisons of the gene expression, the one-way ANOVA test was used to analyze the overall difference in variances between times, and then the *t*-test was performed to compare the difference in means between each pair of times with *p*-values adjusted by the Bonferroni method (**Supplementary Material_ST**). The statistical analyses were done using R 3.4.4 (R Development Core Team, 2018).

## Results

### Identification of *eh-PhoA*^aty^ in *E. huxleyi* and Prediction of Subcellular Localization

**FIGURE 1:**
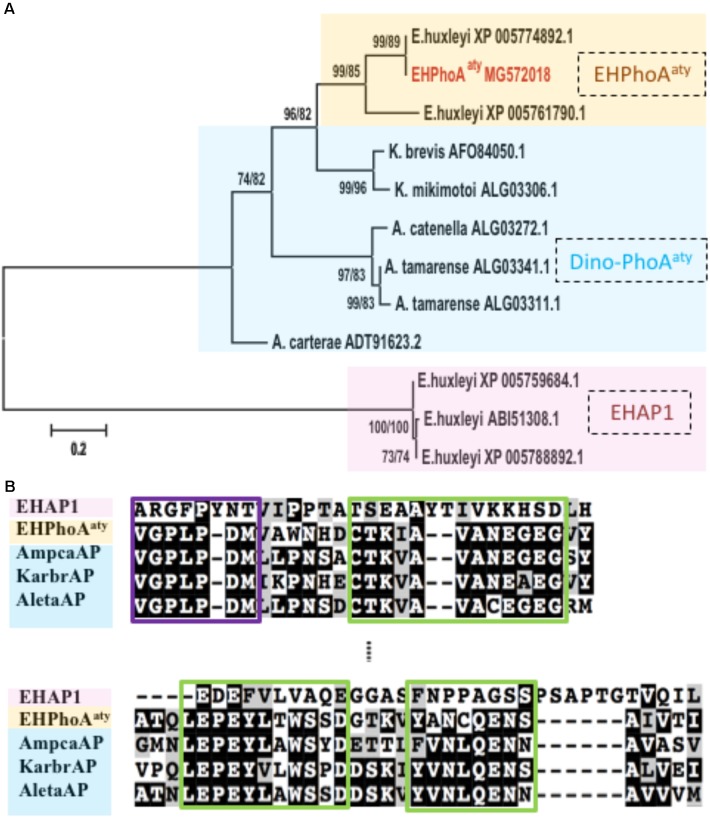
Phylogenetic analysis and conserved regions identified from alignment of amino acid sequences of EH-PhoA^aty^, EHAP1 and dinoflagellate APs. **(A)** Tree topology is shown as a Neighbor Joining tree with 1000 bootstraps and similar topology was obtained using the Maximum-Likelihood. Support value of nodes on each branch are shown as ML/NJ. Color backgrounds indicated the different types of APs. Yellow represents atypical EH-PhoA^aty^ type from *Emiliania huxleyi*, blue represents the PhoA^aty^ type from dinoflagellates (Dino-PhoA^aty^), and pink represents the EHAP1 type from *E. huxleyi*. **(B)** Pairwise comparison of deduced amino acid sequences of EHAP1 (XP 005759684.1), EHPhoA^aty^ (MG572018), AP of dinoflagellate *Amphidinium carterae* (AmpcaAP, ADT91623.2), *Karenia brevis* (KarbrAP, AFO84050.1), *Alexandrium tamarense* (AletaAP, ALG03341.1). Green boxes represent identified conserved domains in PhoA^aty^ (Lin X. et al., 2015).

Four hits (*E*-value < 3e-66; GenBank Accession No: XP_005774892.1, XP_005761790.1, XP_005777715.1, XP_005780497.1) were obtained while using *acaap* to blastx against the *E. huxleyi* CCMP1516 genome (**Supplementary Table 1**). Using degenerate primers designed based on the conserved regions of these sequences, a 634 bp gene fragment was successfully amplified from the cDNA templates of *E. huxleyi* PML B92/11. Sequences of 10 randomly picked clones showed no nucleotide differences and were used to design specific primers for RACE PCR to acquire the full-length ORF region. After assembly, the full-length *eh-PhoA*^aty^ is 2388 bp (GenBank Accession No: MG572018, encoding a protein comprising 696 amino acids). Pairwise sequence comparison confirmed that this gene was 99% identical to a hypothetical protein from *E. huxleyi* CCMP 1516 (GenBank Accession No: XP_005774892.1), which was the top hit in the blast analysis described above (**Supplementary Figure 2**). Phylogenetic analysis showed that, EH-PhoA^aty^ was grouped together with the PhoA^aty^-type of APs identified from dinoflagellates, whilst EHAP1 was on a standalone distant branch (**Figure 1A**). A pairwise sequences comparison of deduced amino acid sequences of EH-PhoA^aty^ with reported PhoA^aty^-type of APs from dinoflagellates, showed that it also contained the conserved domains in PhoA^aty^ (**Figure 1B**). Successful amplification of *eh-PhoA*^aty^ from cDNA template indicated that this gene was actively transcribed in *E. huxleyi* PML B92/11. Furthermore, sequence comparisons showed that *eh-PhoA*^aty^ was different from *ehap1* (Xu et al., 2006) at both the nucleotide (no significant similarity) and amino acid (*E*-value = 1.2, 39% identical) sequence levels, as shown by the distant phylogenetic branch in **Figure 1A**.

The computation model (CELLO) predicted that EH-PhoA^aty^ (**Supplementary Table 2**) was located in the periplasmic compartment (nearly 60% probability), and no signal peptide was identified using computational prediction software packages (**Supplementary Figure 3**). In contrast, the CELLO program predicted that EHAP1 is a periplasmic (nearly 48% probability) or extracellular protein (nearly 25% probability). A signal peptide was found at the N-terminus of EHAP1 (**Supplementary Figure 3**). Moreover, EHAP1 has been experimentally shown to be a secretory protein (Xu et al., 2006).

### Culture Growth and AP Activity Under Different P Conditions

**FIGURE 2:**
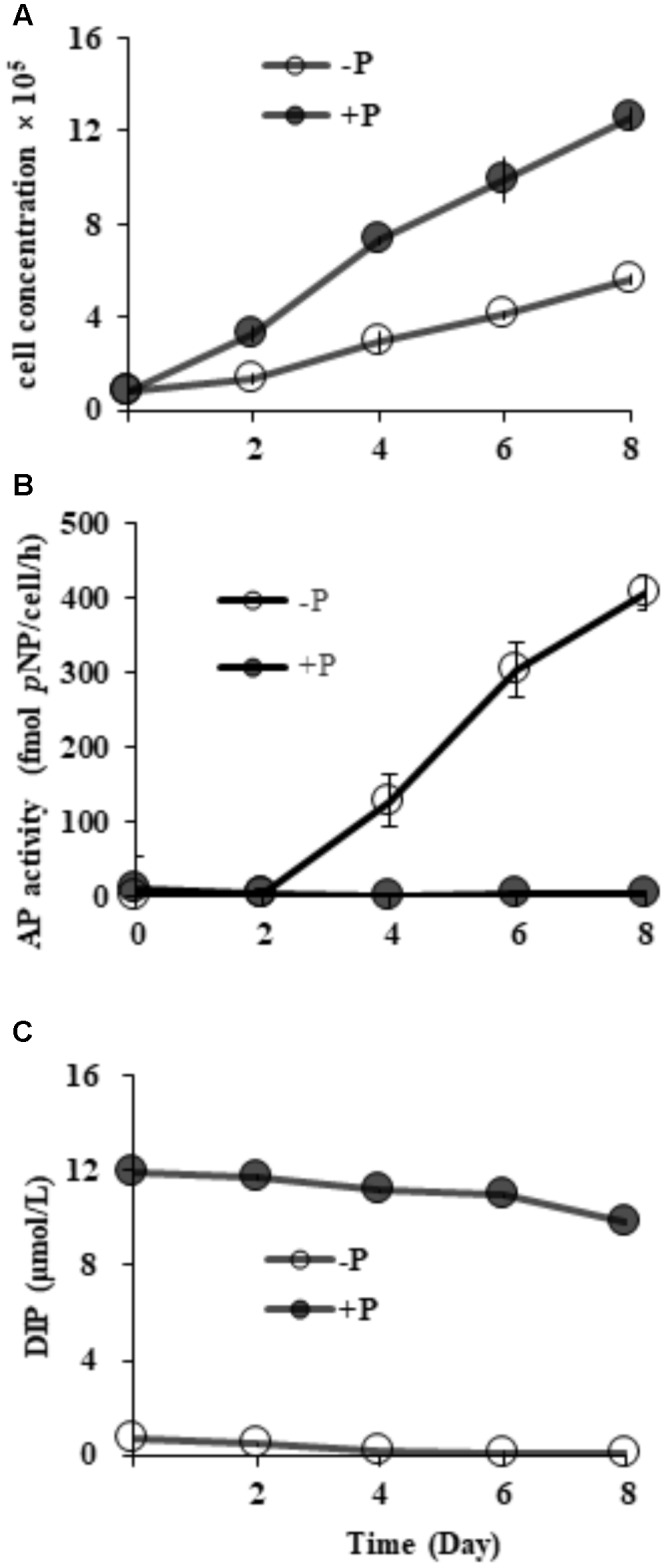
Growth curves **(A)**, AP activity **(B)** and dissolved inorganic phosphorus (DIP) concentrations **(C)** in P-replete and P-depleted cultures. Shown are means ± standard deviations (error bars) from the triplicated cultures.

Starting from the similar initial cell densities, different growth patterns and maximum cell concentrations were observed between the two groups (**Figure 2A**). In the DIP-replete group, cell concentration maintained exponential growth from day 2 to day 8, reaching cell concentration of ∼1.2 × 10^6^ cells mL^-1^ on day 8. Contrastingly, the concentration in the Dip-depleted group was 5 × 10^5^ cells mL^-1^ on day 8, only half of that in the P-replete group.

Compared to barely detectable AP activity in P-replete grown cultures, bulk AP activity in P-depleted cultures increased significantly (*p <* 0.05) along with a decrease in DIP from day 2 (**Figures 2B,C**). AP activity in the P-depleted cultures was about ∼ 127 fmol *p-*NP cell^-1^ h^-1^ on day 4 and reached a maximum of ∼ 405 fmol *p-*NP cell^-1^ h^-1^ in the whole experiment period.

**FIGURE 3:**
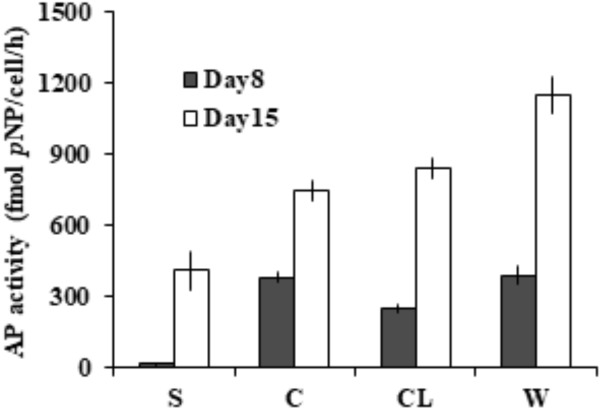
Different subcellular sources of AP activity (intracellular and membrane-associated) in *E. huxleyi* examined on day 8 and day 15. S for supernatant, C for live cells, CL for cell lysate, W for bulk AP activity.

To assess the partition of bulk AP activity into the different subcellular compartments. Further enzymatic activity assays (**Figure 3**) on the cell-free supernatant (S), resuspended cell pellets (C), cell lysate (CL), and bulk culture (W) showed that ∼97% of the measured bulk AP activity was contributed by AP of cell pellets (C) on day 8 and ∼65% on day 15. We also found that the total AP activity of CL was not significantly different from that of C (*p >* 0.05) and the AP activity of the supernatant increased markedly from day 8 to day 15.

**FIGURE 4:**
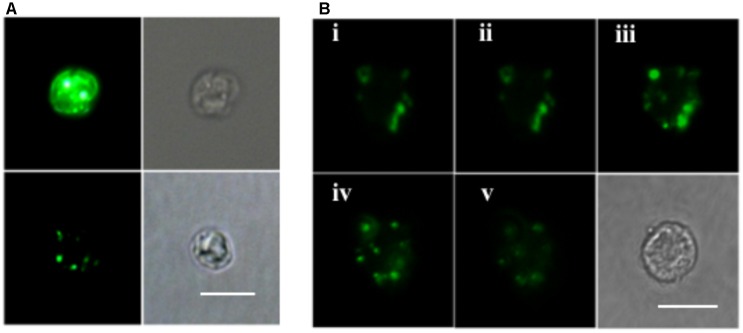
Microscopic images of ELF-97 labeling cells. **(A)** Fluorescent (left) and bright field (right) images of *E. huxleyi* cells grown under P-depleted (upper) and P-replete (bottom) conditions. **(B)** A series of images (i, ii, iii, iv, v) taken with scanning confocal microscope at the depths of 1, 2, 3, 4, 5 μm from P-depleted *E. huxleyi* cells, showing the labeling of AP; Bottom right is a bright field image. Scale bar = 5 μm.

ELF labeling observation was consistent with the above AP activity measurement results. As shown in **Figure 4A**, cells of the P-depleted group displayed stronger green fluorescence compared to the P-replete cells. Confocal microscope images acquired from different layers of the P-depleted cells confirmed that most of the cell-associated AP activity was localized around the cell surface (**Figure 4B**). Low AP activity in the intracellular compartment was in good agreement with the above-mentioned result that AP activity of the CL was similar to that of C. Taken these results together, the major contributor of the bulk AP activity was cell surface associated AP.

### Transcriptional Expression of *eh-PhoA*^aty^ and *ehap1*

**FIGURE 5:**
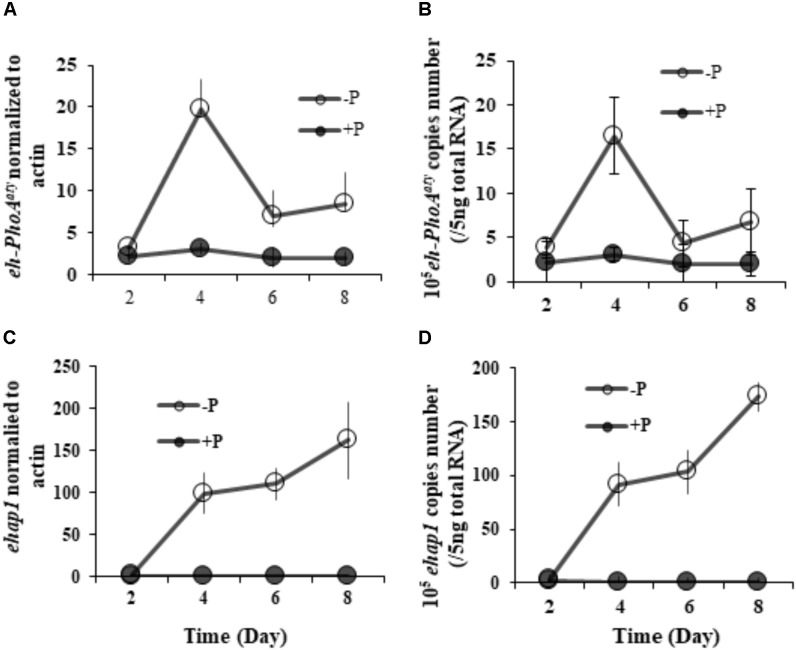
Transcriptional expression of both *ehap1* and *eh-PhoA*^aty^, normalized to actin **(A,C)** and 5 ng total RNA **(B,D)** under P-depleted and P-replete conditions. Solid circles, DIP-replete group; White circles, Dip-depleted group.

RT-qPCR analysis of both *eh-PhoA*^aty^ and *ehap1* showed that expression of both genes was higher in cells grown under P-depleted conditions compared to the P-replete group (*p* < 0.05) (**Figure 5**), regardless of the normalization process. However, each gene expression profile was distinct from each other (**Figure 5**). Under P-depleted conditions, *ehap1* expression was 5–19 fold higher than *eh-PhoA*^aty^, when normalized to actin (**Figures 5A,C**) and about 6–24 fold higher when normalized to the amount of total RNA (**Figures 5B,D**). Secondly, when normalized to actin, *eh-PhoA*^aty^ expression peaked on day 4 (∼6.2 fold higher than day 2, *p* < 0.05, paired *t*-test) and decreased on day 6 (∼3.8 fold lower than day 4, *p* < 0.05, paired *t*-test) and day 8 (∼1.56 fold higher than day 6, *p* > 0.05, paired *t*-test) while the *ehap1* expression increased significantly on day 4 (∼50.5 folds higher than day 2, *p* < 0.05, paired *t*-test) and continued to increase until the end of the experiment (*p* < 0.05, paired *t*-test) (**Figure 5A,C**). Thirdly, a detectable level of *eh-PhoA*^aty^ expression was observed even in P-replete grown cultures, ∼7 fold higher than *ehap1* expression under the same P-replete growth condition (*p* < 0.05) (**Figure 5A,C**).

### Translational Expression of EH-PhoA^aty^ Using Western Blot Analysis

**FIGURE 6:**
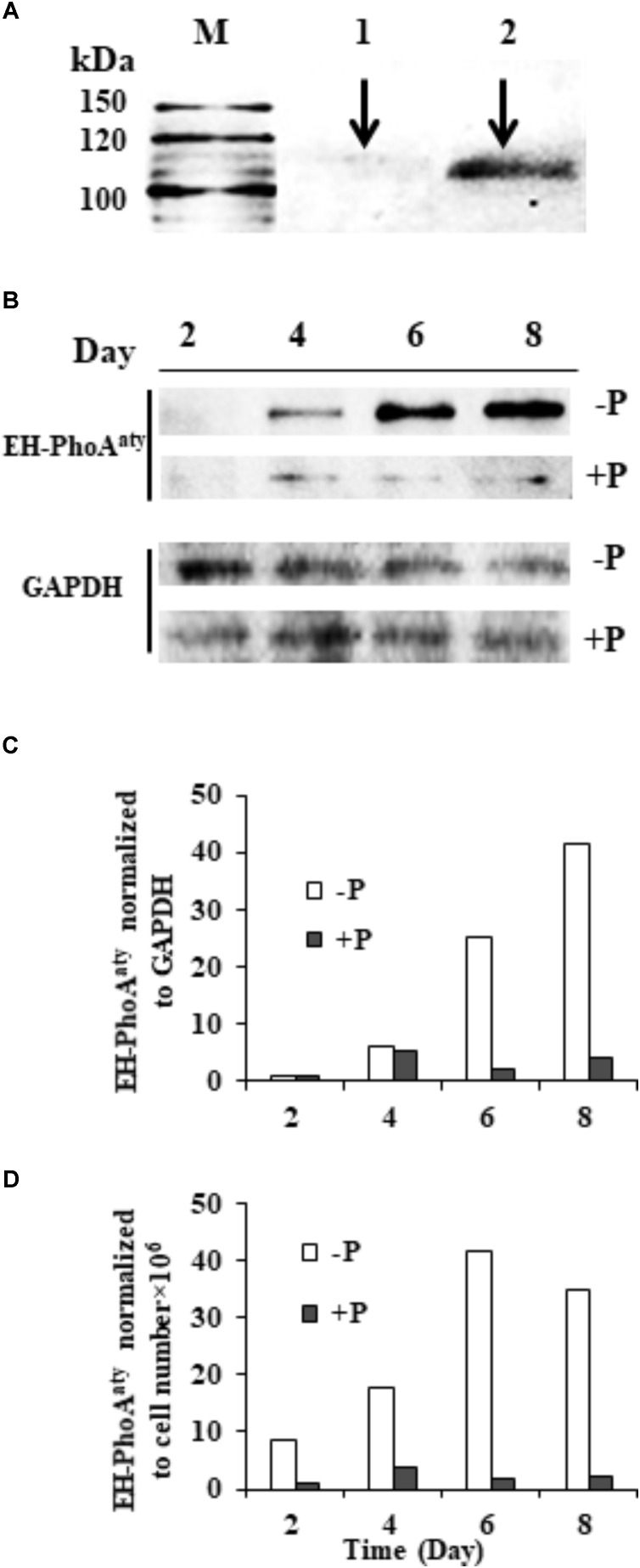
Western blot showing the abundance of EH-PhoA^aty^ under P-depleted and P-replete conditions and competitive immunoblot analysis of EH-PhoA^aty^ in *E. huxleyi*. **(A)** M, marker. Lanes 1 and 2 contained equal amount (10 μg) of *E. huxleyi* total proteins; lane 1, poly-antiserum against AP was pre-incubated with antigen (*p*ACAAP) before the western blot analysis; lane 2, poly-antiserum against AP was pre-incubated with buffer instead. **(B)** Immunoblot images of EH-PhoA^aty^ and GAPDH. **(C,D)** Densitometric analysis of protein EH-PhoA^aty^ normalized to GAPDH and per cell equivalent to the protein loaded into the gels **(B)**.

The affinity and specificity of the antibody used to detect EH-PhoA^aty^ was verified by competitive immunodetection. An aliquot of the antiserum was pre-incubated with antigen (*p*ACAAP). Then, this pre-incubated antibody and the antiserum without pre-incubation with *p*ACAAP were separately used to react with duplicated protein blots of *E. huxleyi*. A protein band of ∼110 KDa was detected on the blot using the antiserum whereas the blot using the pre-incubated antiserum showed that the band was largely eliminated (**Figure 6A**) and the other smaller band (about 50 kD) was slightly eliminated (**Supplementary Figure 4**). To verify that the ∼110 kDa band was the AP being studied, we cut out the bands corresponding to 100–120 kDa and 40–60 kDa for mass spectroscopic analysis. The result showed that EH-PhoA^aty^ was present only in the fragment of 100–120 kD and not in the shorter fragment (**Supplementary Data Sheet 1**). This indicated that the antibody was specific to EH-PhoA^aty^ and the ∼110 kDa band was indeed EH-PhoA^aty^. Also EHAP1 (the predicted MW is 95kD and the experimental size is 75, 110, and 115 kD) (Xu et al., 2010) was also present in the fragment of 100–120 kD. The discrepancy in molecular mass between the detected band (110 kDa) and sequence-based prediction (75 kDa) was probably due to formation of stable dimers or post-translational modification. Some dimers, for instance those linked by sulfide, can remain undissociated in the PAGE gel (Rosen et al., 2010). Besides, N-linked glycosylation can increase the molecular mass of a protein substantially (Kim et al., 2016). In any case, with the antibody of verified specificity, our western blots showed that EH-PhoA^aty^ abundance increased gradually in cells grown under P-depleted conditions, and was markedly more abundant compared to cells grown in the P-replete conditions using Molecular Imager^®^ Chemi Doc XR system for band density analysis (**Figure 6B**). Normalization to GAPDH and equivalent cell numbers gave a similar result (**Figures 6C,D**), with EH-PhoA^aty^ abundance in P-depleted cells on day 8 about 10-fold higher than cells grown under P-replete conditions. The same high P-depleted versus low P-replete pattern was consistently obtained from the triplicate cultures (**Supplementary Figure 1**), although the considerable variation among the triplicate cultures made the difference between their means not statistically different in most of the sample sets.

### Metal Dependency of AP Activity

**FIGURE 7:**
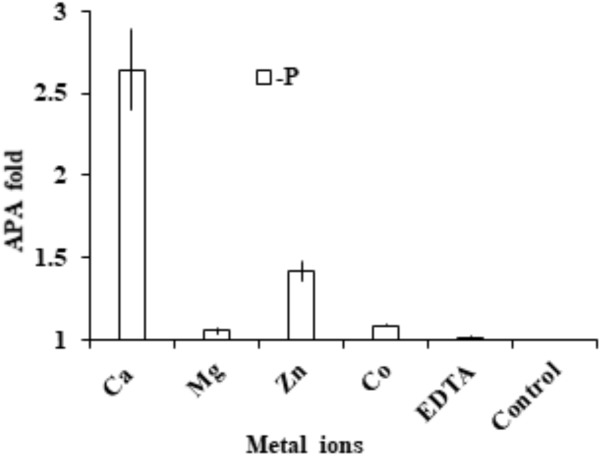
Assay to determine cation cofactors of APs in *E. huxleyi*. After cell lysate was pre-incubated with EDTA to chelate all metal ions, in separate tubes Ca^2+^, Mg^2+^, Zn^2+^, Co^2+^ and EDTA were added and AP was measured and compared to that in the control (no addition of metal or EDTA).

Cells were collected and washed three times with fresh medium to eliminate the left-over activity of the medium. Compared to the EDTA-treated control, we found that AP activity in the group supplied with extra EDTA remained essentially unchanged, indicating that the chelating pre-incubation already completely eliminated AP activity. With this as the basis, we found that the addition of Ca^2+^ and Zn ^2+^ restored AP activity significantly (*p* < 0.05, *t*-test; **Figure 7**). In contrast, the addition of Mg^2+^ or Co^2+^ did not restore AP activity.

## Discussion

### Identification of *eh-PhoA*^aty^ and Occurrence of Two APs in *E. huxleyi*

Many studies have been performed to identify and characterize AP genes in marine microorganisms, leading to the categorization of three types of AP in marine prokaryotes (Gomez and Ingram, 1995; Kriakov et al., 2003; Majumdar et al., 2005). However, relatively few AP genes have been identified in eukaryotic phytoplankton, and most are poorly characterized. Efforts so far indicate that multiple types of AP exist in eukaryotic phytoplankton. For instance, a protein with AP activity was detected in the dinoflagellate *Prorocentrum minimum* (Dyhrman and Palenik, 1997), but a very different family of APs was later identified in a number of dinoflagellate species (Lin et al., 2011, 2012b). The latter APs group phylogenetically with putative AP homologs from other algae, which form a clade recently classified as PhoA^aty^ due to their weak similarity to typical PhoA^EC^ first isolated from *E. coli* (Zalatan et al., 2006) and being distinct from other phosphatases (Lin X. et al., 2015). Here, we identified such a homolog in *E. huxleyi*, with the gene name of *eh-phoA*^aty^. Sequence comparisons and phylogenetic analyses (Lin et al., 2012a) inferred from AP amino acid sequences of eukaryotes indicated that *eh-PhoA*^aty^ is more closely related to atypical dinoflagellate APs than APs from other algae. It is even very different from the other characterized AP in *E. huxleyi ehap1* (Xu et al., 2006), raising the question why does *E. huxleyi* possesses two completely different AP genes?

*Emiliania huxleyi* is a dominant bloom-forming coccolithophore and can be abundant even under oligotrophic conditions (Read et al., 2013). The use of two different APs may be a crucial strategy to exploit P sources under different P availability conditions. An earlier study showed that *E*. *huxleyi* expressed two type alkaline phosphatases, one being constitutive that was synthesized at a steady level under different growth rates and the other being inducible that expressed its highest activity at the lowest growth rate (Riegman et al., 2000). However, the correlations between growth rate and *ehap1* and EH-PhoA^aty^ observed in our study (**Supplementary Figure 5**) suggest that both *ehap1* and EH-PhoA^aty^ are inducible. Yet it is relatively rare in the literature that the same strain or species of eukaryotic algae harbors different alkaline phosphatase genes. In contrast, three prokaryotic APs, PhoA, PhoX, and PhoD, have been found, which share little sequence similarity and possess different subcellular localizations, metal cofactor requirements, and substrates preferences (Luo et al., 2009, 2011; Sebastian and Ammerman, 2009; Kageyama et al., 2011). In the present study, we found that the two types of APs show different expression patterns and our fractionation experiment and computational prediction suggest differential subcellular localizations. All these in part provide insight into the complex utilization of phosphorus in *E. huxleyi*. The ecological implications of the differences between these two AP are discussed below.

### Differential Responses of *eh-PhoA*^aty^ and *ehap1* Expression to P Deficiency and the Ecological Implications

AP activity has been reported to increase in various algae grown under P-limitation, such as dinoflagellates (Lin et al., 2011), cyanobacteria (Tetu et al., 2009), diatoms (Dyhrman and Ruttenberg, 2006), and coccolithophorids (Xu et al., 2006). In the present study, our results showed that AP gene (*eh-PhoA*^aty^ and *ehap1*) expression, protein abundance (EH-PhoA^aty^), and bulk AP activity were induced strongly by P-stress.

At the transcriptional level, the expression of both *eh-PhoA*^aty^ and *ehap1*, relative to the actin reference gene, was higher under P-depleted than P-replete conditions. This indicates that both APs are inducible by P stress. However, the overall expression of these two genes differed considerably. *eh-PhoA*^aty^ gene expression rapidly reached a maximum from day 2 to day 4 before subsequently dropping, whereas *ehap1* expression continued to increase and peaked on day 8 in the whole experiment period (**Figure 5**). It is unclear why *eh-PhoA*^aty^ expression decreased after the initial rapid increase, while corresponding protein levels continued to increase (**Figure 6**), the latter being more consistent with *ehap1* expression levels (**Figure 5**). The discrepancy between gene expression and protein abundance was not likely due to the antibody detecting both EHAP1 and EH-PhoA^aty^, since competitive immunoblotting showed the antibody was specific for EH-PhoA^aty^ although the protein band detected by the antibody in the western blot (110 kDa) was substantially larger in molecular mass than that predicted based on the amino acid sequence of the gene (75 kDa). One possibility is that this EH-PhoA^aty^ forms stable dimers *in vivo*. The other is that EH-PhoA^aty^ is modified, like SUMOylation or glycosylation. These possibilities need to be examined by the sophisticated study of protein structure in the future. Furthermore, *eh-PhoA*^aty^ gene expression (**Figure 5A**) and EH-PhoA^aty^ protein abundance (**Figure 6**) were detected in the P-replete grown cells in which *ehap1* expression was barely detectable (*p* < 0.05) (**Figure 5C**) indicating that some constitutive expression of *eh-PhoA*^aty^ or its expression may be triggered earlier than *ehap1* when phosphate levels in the cell internally decreases.

The difference between *eh-PhoA*^aty^ and *ehap1* was also apparent in their contrasting gene expression profiles. Not only *ehap1* transcript showed continuous increase, but also *ehap1* expression was about 6–20 fold higher than that of *eh-PhoA*^aty^ under P-depleted conditions. If this difference is translated to protein abundance and enzyme activity, EHAP1 would play a more important role than EH-PhoA^aty^ in hydrolyzing phosphoesters for phosphate in *E. huxleyi*.

As demonstrated previously in bacteria (Luo et al., 2009) and eukaryotic phytoplankton (Lin et al., 2012a), APs may also differ in their subcellular localization. EHAP1 was isolated and identified from the medium under P-limitation, and is thus a secretory protein released into the ambient environment (Xu et al., 2006). This is in part supported by computational prediction complemented with the detection of a signal peptide at the N terminus of the protein. In contrast, EH-PhoA^aty^, with a highly diverging sequence compared to EHAP1, has higher possibility as a non-secretory protein supported by computational prediction (periplasmic) and the lack of a signal peptide. For sure, further verification is needed to examine such a prediction, and direct evidence of that would further help us to deduce the ecological implications of these two APs. Further, our AP assays on various cellular components and whole-cell ELF-labeling indicated that the major contributor of AP activity was cell surface located. Consequently, cell-associated AP activity would enable uptake of P from DOP hydrolysis only in the space immediately surrounding algal cells.

### The Cofactor Requirement of APs in *E. huxleyi*

Divalent cations such as Mg^2+^, Ca^2+^, Mn^2+^, Zn^2+^ and Co^2+^ have been reported to be able to activate bacterial or phytoplankton alkaline phosphates (Galperin et al., 1998; Wisniewski, 2006; White, 2009; Sun et al., 2012; Mahaffey et al., 2014). Generally, Zn^2+^ serves as an essential cofactor for PhoA^EC^ (Coleman, 1992; Zalatan et al., 2006). Previous studies indicated that Co^2+^ can replace Zn^2+^ for growth in *E. huxleyi* (Timmermans et al., 2001; Xu et al., 2007) and AP produced by *E. huxleyi* was Zn-dependent and Ca^2+^ could also enhance AP activity (Shaked et al., 2006). Thus, we chose to examine the restoration of AP activity in *E. huxleyi* by the supplement of Ca^2+^, Mg^2+^, Zn^2+^ and Co^2+^ respectively.

After AP activity was eliminated by EDTA, the enzymatic activity could be restored by the supplement of Ca^2+^ (2.6 fold) and Zn^2+^ (1.4 fold) respectively. In addition, our other study found that dinoflagellate AP (PhoA^aty^ type) was also able to restored by Ca^2+^ (Lin et al., in preparation), similar to the widely distributed marine PhoX, which was initially reported in *Vibrio cholerae* (Majumdar et al., 2005) to use calcium and iron as enzyme cofactor (Yong et al., 2014). PhoX has been found to be more widespread in marine bacteria than the conventional PhoA^EC^ in marine environments (Sebastian and Ammerman, 2009) where Zn^2+^ often occurs at subnanomolar concentrations (Moore et al., 2013). Thus, use of Ca^2+^ as a cofactor for AP may be an adaptive response to zinc-P co-limited environments. This would explain in part, from the AP perspective, the cosmopolitan distribution of *E. huxleyi* in both coastal and open oceanic waters. However, direct identification of the co-factor still needs to come from structural analysis of purified AP (Yong et al., 2014), and only then can we start to inquire whether there is differentiation in terms of using Zn^2+^ or Ca^2+^ as the cofactor between EHAP1 and EH-PhoA^aty^.

## Conclusion

We identified a new AP (EH-PhoA^aty^) in *E. huxleyi* PML B92/11, which is similar in protein sequence (42–55% identical) to an atypical eukaryotic type of AP that is widespread in dinoflagellates. Our mRNA and protein quantification results showed that the expression of both *eh-PhoA*^aty^ gene and EH-PhoA^aty^ protein were inducible by P deficiency in *E. huxleyi*. Different transcriptional expression profiles between *eh-PhoA*^aty^ and *ehap1*, suggest low level constitutive expression of *eh-PhoA*^aty^ or a differential P stress threshold triggering their expression when phosphate levels in the cell internally decrease. Furthermore, we found that Ca^2+^ can highly restore cell associated AP activity suggesting an adaptation to zinc-P co-limited open ocean environments. However, further work needs to resolve their potentially different modes of action and the cofactor requirement of EH-PhoA^aty^ and EHAP1.

## Author Contributions

SL and XL designed the study. TL conducted the experiments. CG, YZ, and CW contributed to the experimental design and data analysis. TL, SL, XL, and CW wrote the paper.

## Conflict of Interest Statement

The authors declare that the research was conducted in the absence of any commercial or financial relationships that could be construed as a potential conflict of interest.

## Footnotes

1 https://embnet.vital-it.ch/software/BOX_form.html
