# Supplementary material for: Identification and Expression Analysis of an Atypical Alkaline Phosphatase in Emiliania huxleyi
Source: Front Microbiol. 2018 Sep 19;9:2156. doi: 10.3389/fmicb.2018.02156 (PMC6156274; doi:10.3389/fmicb.2018.02156)
Supplement: Supplementary file 3 [file Image_1.PDF]

Supplementary Figures:

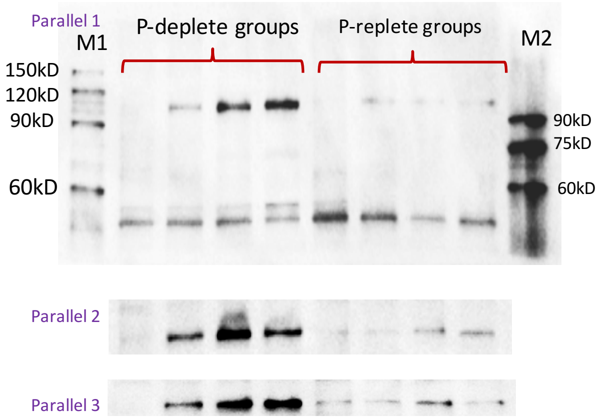

Supplementary Fig 1. The full image acquired from western blot analysis, shown as three-time repetition.

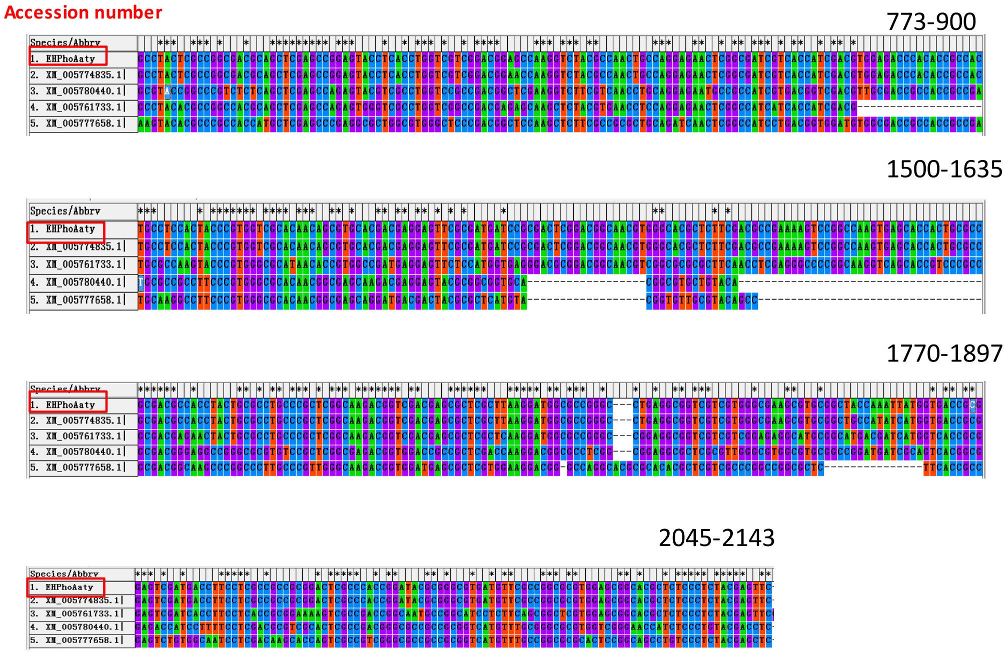

Supplementary Fig 2. The alignment of four hits (Supplementary Table 1) compared with the full-length ORF *eh-phoA*^{aty} (marked with red square), right upper numbers of each line represent the nucleotide site range of *eh-phoA*^{aty}**.** XM_005774835.1 (XP_005774892.1); XM_005761733.1(XP_005761790.1); XM_005780440.1 (XP_005780497.1); XM_005777658.1 (XP_005777715.1).

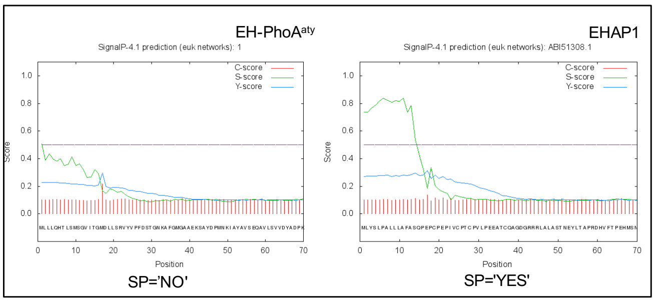

Supplementary Fig 3. Identification of signal peptide of EH-PhoA^{aty} and EHAP1 using SignalP V4.1.

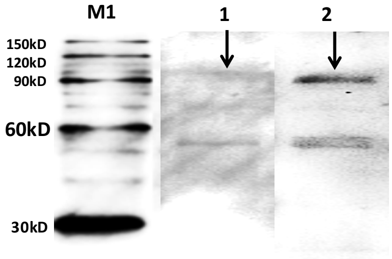

Supplementary Fig 4. Competition for the epitope. M, marker. Lanes 1 and 2 contained equal amount (10 μg) of *E. huxleyi* total proteins; lane 1, poly-antiserum against AP was pre-incubated with antigen (*p*ACAAP) before the western blot analysis; lane 2, poly-antiserum against AP was pre-incubated with buffer instead.

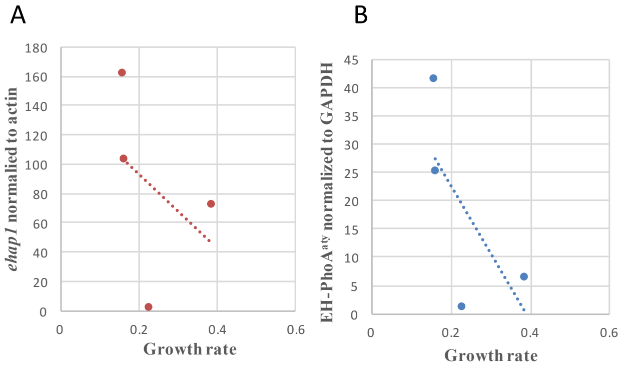

Supplementary Fig 5. The correlations between growth rate and *ehap1* (A) and EH-PhoA^{aty} (B).

Supplementary Tables:

Supplementary Table 1. Four hits acquired from blastx against *E. huxelyi* CCMP 1516 genome using *acaap* as query, AP gene identified in dinoflagellate *A. carterae* (Lin et al., 2011).

GenBank accession number	Protein function	E value	query	Identify
XP_005774892.1	hypothetical	2e-151(5e-41)*	88%(92%)*	45%(49%)*
XP_005761790.1	hypothetical	2e-117(3e-28)*	88%(97%)*	41%(42%)*
XP_005780497.1	hypothetical	3e-66(1e-24)*	58%(90%)*	41%(41%)*
XP_005777715.1	hypothetical	5e-69(9e-23)*	90%(87%)*	32%(40%)*

* represent the value of using *p*ACAAP as query to blastp against *E. huxelyi* CCMP 1516 genome.

Supplementary Table 2. Predicted subcellular localizsation of APs in *E. huxleyi* using CELLO.

	CELLO
EH-PhoA^{aty}	Periplasmic (~59% probability)
EHAP1	Periplasmic (~48% probability)<br>Extracellular (~25% probaility)
